# Lecture recording, microlearning, video conferences and LT-platform – medical education during COVID-19 crisis at the Medical University of Graz

**DOI:** 10.3205/zma001407

**Published:** 2021-01-28

**Authors:** Josef Smolle, Andreas Rössler, Herwig Rehatschek, Florian Hye, Sabine Vogl

**Affiliations:** 1 Medizinische Universität Graz, Institut für Medizinische Informatik, Statistik und Dokumentation, Graz, Austria; 2 Medizinische Universität Graz, Otto-Loewi-Forschungszentrum, Lehrstuhl für Physiologie, Graz, Austria; 3 Medizinische Universität Graz, Stabsstelle Lehre mit Medien, Graz, Austria; 4 Medizinische Universität Graz, Studium und Lehre, Graz, Austria

**Keywords:** E-Learning, COVID-19 crisis, microlearning, video conferencing, lecture recording, AdInstruments

## Abstract

**Objective:** In the course of the COVID-19 crisis it became necessary to convert the majority of classroom teaching to e-learning. This should be done in a uniform and transparent way for the study programs of the Medical University of Graz.

**Methodology: **We built on the Virtual Medical Campus, which has existed since 2003. For the summer semester 2020, we focused on an expansion of the automatic lecture recording system, microlearning and the implementation of video conferences as well as the learning platform LT.

**Results: **The number of lecture recordings increased from 170 to more than 700, weekly accesses reached more than 80,000, with nearly 4,200 students. In the Microlearning system, an average of 82,516+-12,071 SEM learning steps per week were completed, which represented a highly significant increase compared to the same period of the previous year (15,101+-4,278 SEM; t-test: t=5,2638, p<0,0001). Video conferencing via WebEx was a newly introduced tool that was used extensively for interactive seminars, but also for oral exams. The LT platform from AdInstruments was successfully used as a replacement for practical training, especially in physiology.

**Conclusions: **Based on sufficient preparatory work, a rapid expansion of e-learning ensured that teaching could be continued in the form of home learning despite the exceptional situation caused by COVID-19. Success factors were the provision of selected technical tools, consistent communication of the university management and technical and content support for teachers and students by a central staff unit.

## Zusammenfassung

**Zielsetzung: **Im Zuge der COVID-19-Krise wurde es notwendig, den Großteil des Präsenzunterrichts auf E-Learning umzustellen. Dies sollte für die Studienrichtungen der Medizinischen Universität Graz in möglichst einheitlicher und transparenter Form erfolgen.

**Methodik: **Wir bauten auf dem seit 2003 bestehenden Virtuellen Medizinischen Campus auf. Für das Sommersemester 2020 setzten wir auf einen Ausbau des automatischen Vorlesungsaufzeichnungssystems, des Microlearnings und auf die Implementierung von Videokonferenzen sowie der Lernplattform LT.

**Ergebnisse: **Die Zahl der Vorlesungsaufzeichnungen stieg von 170 auf mehr als 700, die wöchentlichen Zugriffe erreichten mehr als 80.000, bei knapp 4.200 Studierenden. Im Microlearning-System wurden durchschnittlich 82.516+-12.071 SEM Lernschritte pro Woche absolviert, was gegenüber dem Vergleichszeitraum des Vorjahres eine hoch signifikante Steigerung darstellte (15.101+-4.278 SEM; t-Test: t=5.2638, p<0.0001). Videokonferenzen via WebEx waren ein neu eingeführtes Werkzeug, das flächendeckend für interaktive Seminare, aber auch für mündliche Prüfungen, verwendet wurde. Als Ersatz für Praktika wurde vor allem in der Physiologie die LT-Plattform von AdInstruments erfolgreich eingesetzt.

**Schlussfolgerungen: **Aufbauend auf ausreichenden Vorarbeiten konnte durch einen raschen Ausbau des E-Learnings ein entsprechender Lehrbetrieb trotz COVID-19-bedingter Ausnahmesituation in Form von Home Learning gewährleistet werden. Erfolgsfaktoren waren die Bereitstellung von ausgewählten technischen Werkzeugen, konsistente Kommunikation der Universitätsleitung und eine technisch-inhaltliche Unterstützung der Lehrenden und Studierenden durch eine zentrale Stabsstelle.

## 1. Introduction

When there was an extensive lockdown in Austria in April 2020, we were in the positive position of not having to start virtualization from scratch. The development of e-learning was already initiated in 2002 with government subsidies [1], was taken into regular operation, and was further expanded through subsequent funding tranches and from the university’s global budget. As a special feature, our university – challenged by an abrupt change in the legal framework – held the first semester for students of human and dental medicine purely virtually in 2005/06 [2].

At the time of the changeover to Home Learning, we had a Moodle-based comprehensive e-learning portal (VMC - Virtual Medical Campus) with more than 13,000 learning objects. For the period of extensive virtualization, we subsequently relied on four components: Lecture recordings, microlearning and video conferencing and the use of the learning platform LT as a replacement for practical training. Thus, the presentation refers to the special conditions of Med Uni Graz with the relevant previous experience, which may be specifically different at other locations. In addition, a priori attention was paid to the quick and easy implementation, whereby the didactic requirements of the respective subject areas were taken into account when selecting the mentioned formats. 

## 2. Lecture recordings as a low-threshold offer for the lecturers

In the course of the new construction of the preclinical institutes on the Med Campus, five lecture halls were equipped with a lecture recording system based on Epiphan hardware [https://www.epiphan.com/]. This is permanently installed and always records two streams: Lecturer and PC output. In the course of the corona crisis, all major lectures were cancelled and made available as video recordings instead. We deliberately refrained from live streaming for the major lectures because interaction plays only a limited role even in face-to-face classes and live streaming would result in a loss of time independence for the students. For video recording, the teachers came alone into the lecture halls, gave their lectures and were each technically supported by one person. Subsequently, a professional post-processing and the provision of the finished lectures took place both on the video portal VITAL [https://vital.medunigraz.at] of the Med University of Graz and linked in the eLearning platform Moodle. In view mode, students can choose between pure presentation view, pure lecturer view and the – usual – double view. While 170 lectures were available in the system until the corona lockdown, after a largely virtual summer semester 2020, the number of lectures is now 743. 4,000 lectures are accessed on average each week, reaching peak values of up to 8,556 with almost 4,200 students.

## 3. Microlearning as interactive learning support

Microlearning is learning in small steps, independent of time and place, preferably via mobile devices [3]. Our university has licensed the microlearning software KnowledgeFox (KnowledgeFox GmbH, Vienna) since 2017. The microlearning content consists of knowledge cards (information cards, single select, multiple select, cloze text or vocabulary cards), which contain a meaningful explanation and, if necessary, pictures and videos. The underlying repetition algorithm uses the “power law of practice” [4], the “testing effect” [5] and the “spacing effect” [6]. By the end of the summer semester 2020, the microlearning system comprised approximately 20,000 knowledge cards. The number of weekly knowledge card calls increased from 15,101+-4,278 SEM in the summer semester 2019 to 82,516+-12,071 SEM in the summer semester 2020 (t-test: t=5,2638, p<0.0001). It is noteworthy that two courses in physiology, which were only set up during the semester, achieved more than 160,000 and 180,000 learning steps respectively in a very short time, which can be attributed both to the quality of the courses offered and to the stringent integration into the teaching and examination process. 

## 4. Video conferences

For several weeks it was not permitted to hold face-to-face meetings. In order to be able to guarantee interactive teaching nevertheless, the University licensed Webex (Meetings, Teams and Trainings) (Cisco Corp., San Jose, USA) and made it available to all teachers and students. The staff unit Teaching with Media offered the teachers information material, detailed instructions and personal training as well as active practice units in order to become familiar with the system. The teachers used the tool for live streaming of their presentations and interactive discussion. In addition to holding seminars, this tool was also used for oral examinations.

## 5. LT learning platform as a replacement for internships

Since the lack of classroom teaching meant that no practical training could be held, LT (AdInstruments) was used as an online platform [7]. Especially in the field of physiology, examples were worked out, with the help of which students could make observations on themselves at home, enter them into an online protocol and finally perform an online final test. The experimental proof of the blind spot in the eye in the context of sensory physiology is one such example of a self-experiment. More than 97% of the students (476 of 488) made use of LT and all of them also completed the corresponding final online tests.

The changes in the use of different e-learning formats before and during the corona crisis are shown semi-quantitatively in table 1 (Tab. 1). 

## 6. Outlook

With the four central tools – lecture recordings, microlearning as interactive learning support, video conferencing for holding seminars and exams and LT as a replacement for practical training, Med Uni Graz has succeeded in bridging the lockdown caused by corona and continuing to offer students lessons without interruption. It was a great advantage that e-learning as a form of teaching/learning did not have to be taken up again, but was already well established on campus and only required consistent upscaling. The decisive factors for success are a concentration on a few tools, concise communication by the university management, close coordination of those involved, support of the teaching staff by a central e-learning unit and the commitment of the teaching staff. The digital skills and digital equipment of the students, which – in contrast to the “virtual semester” in the academic year 2005/2006 – are taken for granted today, posed no challenge. In the following, however, we will deal with the differentiated didactic evaluation of the various methods of knowledge transfer and the advantages and disadvantages from the perspective of students and teachers. For example, we are preparing a survey on acceptance among teachers and students as well as comparative studies of examination performance after the elearning phases compared to the attendance phases [8]. 

We assume that the expansion of e-learning offerings as an accompanying teaching and learning tool will be maintained beyond the corona crisis and that it will be possible to react quickly and efficiently in the event of a new pandemic challenge. In particular, we believe that this will affect both lecture recordings and the use of video conferencing, although the latter was hardly used at all in our company before the crisis and has now found high acceptance. The same applies to microlearning, which was further developed during the lockdowns and will continue to be made available to students and expanded in the future.

## Competing interests

The authors declare that they have no competing interests. 

## Figures and Tables

**Table 1:**
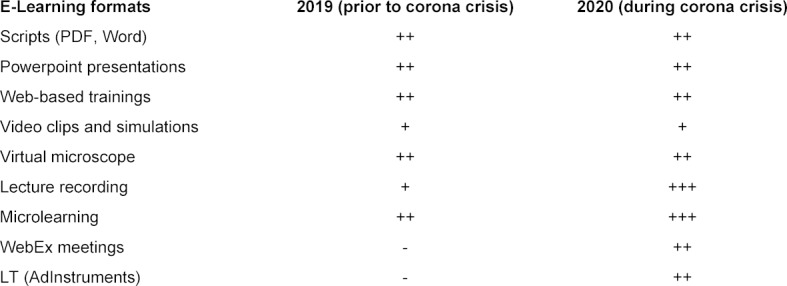
Utilization of e-learning formats prior and during the corona crisis (semiquantitative assessment).

## References

[1] Smolle J, Staber R, Jamer E, Reibnegger G, Tavangarian D, Nölting K (2005). Aufbau eines universitätsweiten Lern- Informationssystems parallel zur Entwicklung innovativer Curricula - zeitliche Entwicklung und Synergieeffekte. Auf zu neuen Ufern - E-Learning heute und morgen.

[2] Smolle J, Neges H, Staber R, Macher S, Reibnegger G, Seiler Schiedt E, Kälin S, Sengstag C (2006). Virtuelles Eingangssemester im Studium der Humanmedizin. Kontext, Nutzung, Ergebnisse. E-Learning - alltagstaugliche Innovation?.

[3] Bruck PA, Motiwalla L, Foerster F (2012). Mobile Learning with micro-content: a framework and evaluation.

[4] Evans NJ, Brown SD, Douglas, Mewhort DJ, Heathcote A (2018). Refining the law of practice. Psychol Rev.

[5] Larsen DP, Butler AC, Roediger HL (2008). Test-enhanced learning in medical education. Medl Educ.

[6] Dempster FN (1988). The spacing effect: A case study in the failure to apply the results of psychological research. Am Psychol.

[7] Marcondes FK, Cardozo LT, Luchi KCG, Irfannuddin M, Karatzaferi C, Rocha MJ, Carroll RG (2018). Meeting report: IUPS and ADInstruments 2017 Teaching Workshop. Adv Physiol Educ.

[8] Nicoll P, MacRury S, van Woerden, HC, Smyth K (2018). Evaluation of technology-enhanced learning programs for health care professionals: systematic review. J Med Internet Res.

